# Roles of p38 MAPK signalling in intervertebral disc degeneration

**DOI:** 10.1111/cpr.13438

**Published:** 2023-03-05

**Authors:** Zheng‐wei Shi, Lei Zhu, Zong‐rang Song, Tuan‐jiang Liu, Ding‐jun Hao

**Affiliations:** ^1^ Department of Spine Surgery, Hong‐Hui Hospital Xi'an Jiaotong University College of Medicine Xi'an China

## Abstract

Intervertebral disc degeneration (IVDD) is a common degenerative disease mediated by multiple factors. Because of its complex aetiology and pathology, no specific molecular mechanisms have yet been identified and no definitive treatments are currently available for IVDD. p38 mitogen‐activated protein kinase (MAPK) signalling, part of the serine and threonine (Ser/Thr) protein kinases family, is associated with the progression of IVDD, by mediating the inflammatory response, increasing extracellular matrix (ECM) degradation, promoting cell apoptosis and senescence and suppressing cell proliferation and autophagy. Meanwhile, the inhibition of p38 MAPK signalling has a significant effect on IVDD treatment. In this review, we first summarize the regulation of p38 MAPK signalling and then highlight the changes in the expression of p38 MAPK signalling and their impact on pathological process of IVDD. Moreover, we discuss the current applications and future prospects of p38 MAPK as a therapeutic target for IVDD treatment.

## Graphical abstract

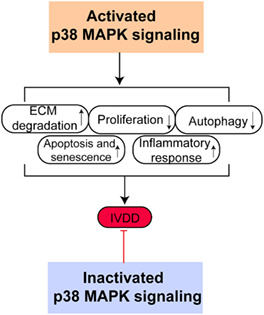

## 1 INTRODUCTION

Low back pain (LBP) is a widespread health problem that is a leading contributor to disability as well as the global burden of disease.^1^ Intervertebral disc (IVD) degeneration (IVDD) is a common degenerative musculoskeletal disease that is regarded as the main cause of LBP. Normal IVD consists of avascular tissue and is primarily composed of an outer annulus fibrosus (AF), an internal nucleus pulposus (NP) and cranially and caudally cartilaginous endplates (CEPs).^2^, ^3^ Due to ageing, mechanical overloading, nutritional deficiency, oxidative stress and other factors, the above‐mentioned structures can exhibit pathological changes and loss of biological function, including reduction in proteoglycan and water content, the decline in IVD height, endplate sclerosis and osteophyte formation and decreased ability to withstand compression load.^3^, ^4^, ^5^ IVDD progression may lead to spinal degenerative diseases, including disc herniation, spinal canal stenosis, spondylolisthesis and degenerative scoliosis, which can cause pain and disability.^6^ Although IVDD has been studied to a certain extent in recent years, the underlying mechanisms remain unclear, and effective treatment approaches are not available. Therefore, the need to explore the underlying mechanisms and develop novel therapeutic approaches for the early recovery of IVDD is a matter of urgency.

Mitogen‐activated protein kinases (MAPKs) are serine and threonine (Ser/Thr) protein kinases that convert extracellular stimuli into a broad range of cellular responses. MAPKs are highly conserved regulatory mechanisms in mammals and are divided into three main groups, namely extracellular signal‐regulated kinases (ERKs), c‐Jun N‐terminal kinases (JNKs) and p38 isoforms (α, β, γ and δ).^6^, ^7^ As a member of the MAPK family, p38 MAPK signalling is involved in multiple diseases, such as inflammatory,^8^ cardiovascular^9^ and neurodegenerative diseases,^10^ as well as cancer.^11^ Meanwhile, a growing body of evidence indicates that p38 MAPK signalling is a suitable therapeutic target and a potential treatment strategy for several diseases.^12^, ^13^, ^14^

For the past few years, p38 MAPK signalling has received considerable attention in the field of IVD research. An increasing number of studies have shown that dysregulation of p38 MAPK signalling is associated with IVDD progression, including IVD cell death, inflammatory response, imbalance of the extracellular matrix (ECM) homeostasis and other cell phenotypes.^15^, ^16^, ^17^ Simultaneously, targeting p38 MAPK signalling has shown promising therapeutic potential in alleviating IVDD. In the following sections, we will focus on describing the regulation of p38 MAPK signalling, changes in its expression and its role in the process of IVDD. Moreover, we will discuss the approaches for regulating p38 MAPK signalling as a treatment strategy for IVDD.

## 2 p38 MAPK SIGNALLING

p38 MAPK is a class of evolutionarily conserved MAPKs that transduce extracellular signals to regulate multiple cellular processes. The p38 MAPK family has four isoforms, consisting of p38α (MAPK14), p38β (MAPK11), p38γ (MAPK12) and p38δ (MAPK13), which are encoded by distinct genes.^18^, ^19^ Among the four isoforms, sequence identity is conserved within the kinase domains, and there is sequence homology. For example, p38α shares 75% sequence identity with p38β and 60% with p38γ and p38δ. In addition, p38γ and p38δ have approximately 70% similarity.^20^, ^21^ Despite their high sequence homology, these isoforms have notable differences in tissue expression, sensitivity to chemical inhibitors and cellular functions. For example, p38α and p38β are ubiquitously expressed in most of cells and tissues, whereas p38γ and p38δ have more restricted expression patterns.^22^ Significantly, the four isoforms have different sensitivity to chemical inhibitors, such as SB203580.^21^ In addition, because genetic ablation of p38α (MAPK14) results in embryonic lethality in mice, p38α has been proven to be the only p38 kinase essential for mouse embryo development.^23^ However, ablation of other isoforms, or disruption of single p38δ and p38γ, does not result in major abnormalities in live mice.^24^, ^25^ Overall, current evidence suggests that p38α and p38β act together in heart development, sex determination, mitotic entry inhibition and induction of T‐cell immunity.^26^, ^27^, ^28^, ^29^ p38γ and p38δ can cooperate in tissue regeneration and immune responses in certain cell lines.^30^ Interestingly, some research has shown that a lack of p38α can promote the activation of p38γ or p38δ.^31^, ^32^ Taken together, based on the complex relationship between the isoforms, the biological functions of this intricate signalling pathway require further exploration.

## 3 REGULATION OF p38 MAPK SIGNALLING

**FIGURE 1:**
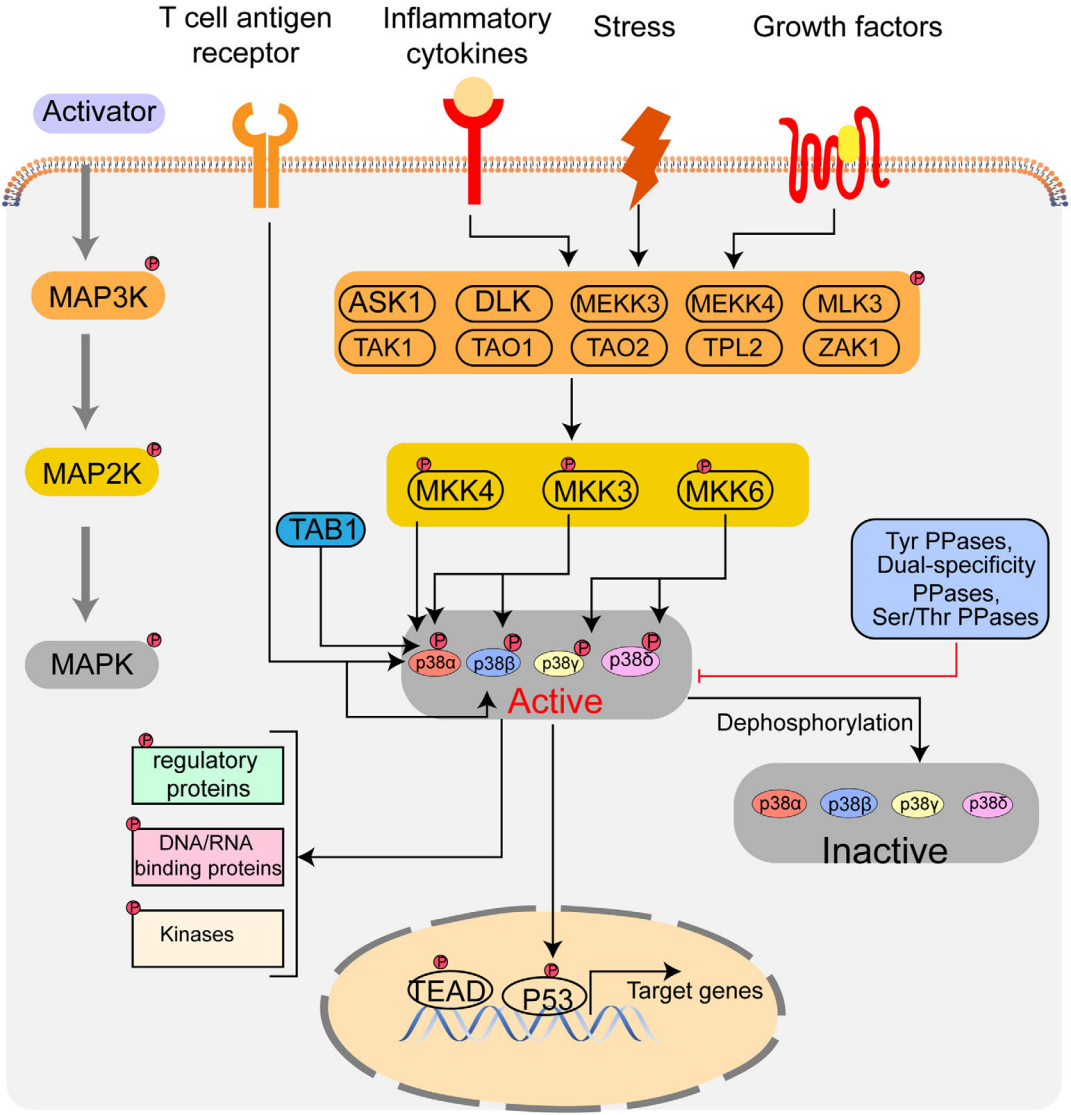
Regulation of p38 MAPK signalling. The activation of p38 MAPK is mainly associated with the MAPK kinase kinase (MAP3K)–MAP kinase kinase (MKK) pathway. First, extracellular stimuli can activate MAP3Ks. Then, the activation of MAP3Ks phosphorylates MAP2Ks, including MKK3, MKK4 and MKK6. Activated MKK4 specifically promotes the activation of p38α. Phosphorylation of MKK3 can activate both p38α and p38β, and phosphorylation of MKK6 is responsible for phosphorylating p38γ and p38δ. TAB1 (TAK1‐binding protein 1) can directly interact with p38α. Furthermore, p38α and p38β can also be activated by the T cell antigen receptor signalling pathway. Activated p38 MAPK can be dephosphorylated and thereby deactivated by protein phosphatases, such as dual‐specificity phosphatases, serine/threonine phosphatases and tyrosine phosphatases.

In mammals, p38 MAPK can be activated by many extracellular stimuli such as oxidative and osmotic stress, ultraviolet radiation, hypoxia, ischaemia, interleukin‐1 (IL‐1) and tumour necrosis factor‐alpha (TNF‐α).^19^ Activated p38 MAPK regulates embryonic development, immune responses, cell cycle, endocytosis, metabolism and cytoskeleton dynamics by phosphorylating downstream substrates.^33^ Generally, the activation of p38 MAPK is associated with the MAPK kinase kinase (MAP3K)–MAP kinase kinase (MKK) pathway (Figure 1). First, extracellular stimuli, such as oxidative stress and cytokines can activate MAP3Ks via phosphorylation or by promoting their interaction with the Ras homologous (Rho) protein, cell division control protein 45 (CDC45) and Rac small GTPases. Next, the activation of MAP3Ks can phosphorylate MAP2Ks, including MKK3, MKK4 and MKK6. Notably, among active MAP3Ks, only apoptosis signal‐regulating kinase 1 (ASK1) can activate MKK4 that specifically promotes the activation of p38α.^34^ Moreover, the phosphorylation of MKK3 activates p38α and p38β together, and the phosphorylation of MKK6 is responsible for the activation of p38γ and p38δ.^35^, ^36^ In addition to the above regulatory pattern, activation of p38α can also depend on the autophosphorylation of p38α. This activation mechanism is independent of MAP2K. Mechanistically, TAK1‐binding protein 1 (TAB1) can directly interact with p38α and promote autophosphorylation on Thr 180 and Tyr 182, resulting in full activation of p38α.^37^ Furthermore, p38α and p38β can also be activated by the T cell antigen receptor (TCR) signalling pathway in addition to the MAP2K‐dependent mechanism.^38^ Overall, phosphorylated p38 MAPK is located in the cytoplasm and nucleus, which, in turn, can activate a series of substrates, such as transcription factors, protein kinases and cytosolic and nuclear proteins.

Contrarily, the activated p38 MAPK can be dephosphorylated and thereby deactivated by protein phosphatases such as dual‐specificity phosphatases, serine/threonine phosphatases and tyrosine phosphatases.^39^ A series of post‐translational modifications can also mediate the deactivation of p38 MAPK, including ubiquitination, acetylation and methylation.^40^ Moreover, one study showed that the phosphorylation of p38α can negatively regulate the expression of the p38γ protein via the ubiquitin‐proteasome pathways.^32^ This result represents a novel mechanism by which p38 MAPK isoforms can be regulated by each other. Taken together, the regulation of p38 MAPK is associated with multiple regulatory mechanisms. Therefore, identifying specific mechanisms that are responsible for this regulation in certain cellular processes is a great challenge.

## 4 EXPRESSION CHANGES OF p38 MAPK IN IVDD

Current evidence indicates that the expression level of p38 MAPK changes with the degree of IVDD and shows a significant difference in NP versus AF tissue. Yang et al.^41^ first found that the expression level of p38 MAPK in the NP tissue was higher than that in the AF tissue. Moreover, the expression levels of the four p38 MAPK isoforms also showed a considerable difference. The expression levels of p38α and p38β were remarkably higher in the IVDD tissue compared with that in the normal NP tissue, while the expression level of p38δ was lower in the IVDD tissue compared with that in the normal NP tissue and could only be detected in half of the IVDD tissue; further, the expression level of p38γ was lower than that of p38δ. In vivo, Dai et al.^42^ showed that the expression level of the activated p38 MAPK was significantly higher in the rat IVDD groups compared with the control group (*p* < 0.01). Cheng et al.^43^ found a significant increase in the expression level of activated p38 in degenerated rat NP tissues. In addition, a series of reports confirmed that the expression level of activated p38 was higher in degenerative NP cells than that in normal NP cells. From the above results, we can conclude that p38 MAPK may play a more important role in the NP tissue rather than in the AF tissue. In contrast, though the expression of p38α and p38β is higher than that of p38δ and p38γ, there is still no specific evidence as to which whether p38α or p38β is mainly responsible for the progress of IVDD. Therefore, it is necessary to further explore the individual functions of p38α and p38β in IVDD progression.

## 5 ACTIVATING p38 MAPK SIGNALLING CONTRIBUTES TO IVDD

**FIGURE 2:**
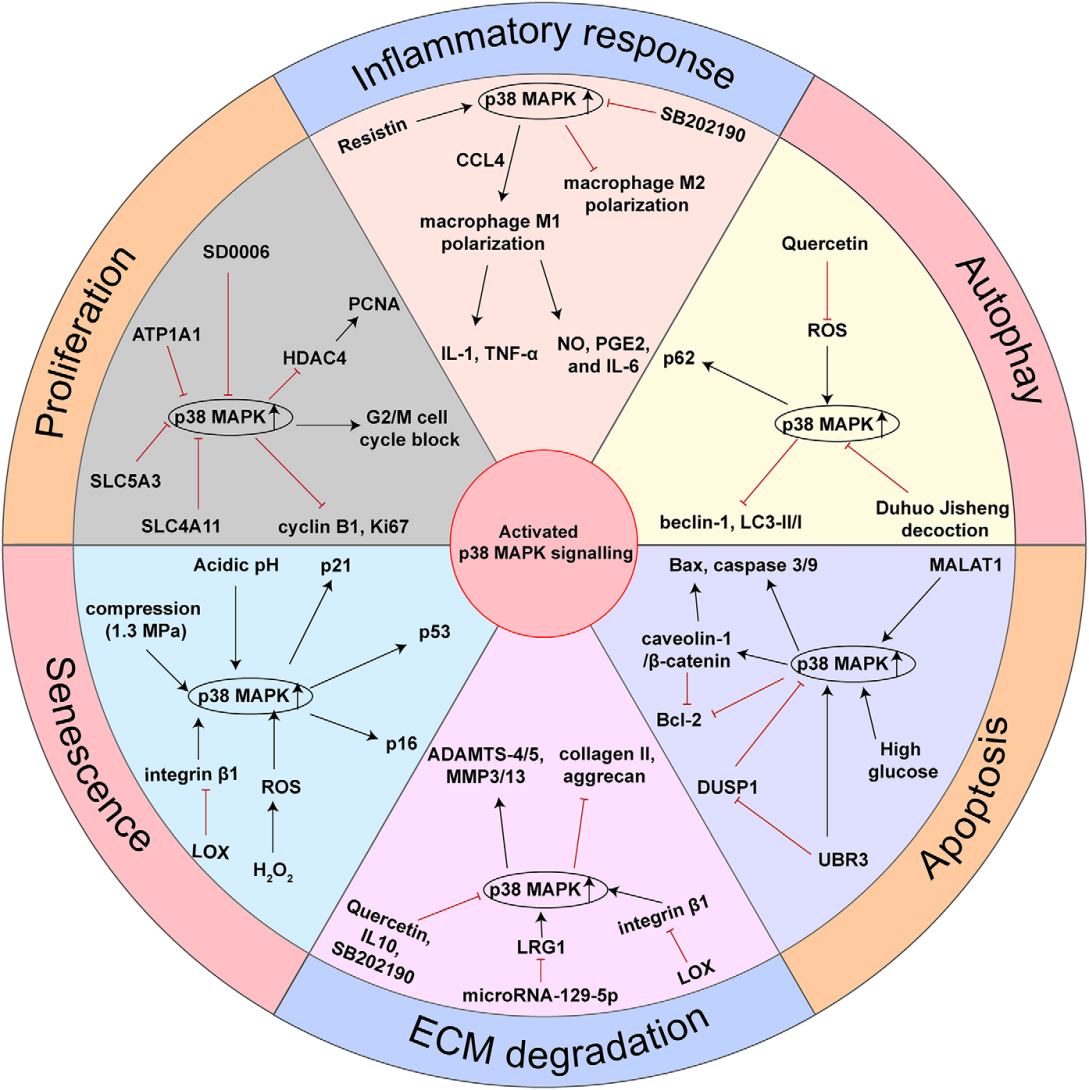
Functions of activated p38 MAPK signalling in the progression of intervertebral disc degeneration (IVDD). The activation of p38 MAPK signalling is associated with the progression of IVDD, including mediating the inflammatory response, increasing extracellular matrix (ECM) degradation, promoting cell apoptosis and senescence and suppressing cell proliferation and autophagy.

As mentioned previously, p38 MAPK activation is involved in IVDD progression. Meanwhile, increasing evidence has suggested that activated p38 MAPK is associated with the pathophysiological characteristics of IVDD, including the inflammatory response, ECM degradation, IVD cell apoptosis, proliferation and senescence and level of autophagy (Figure 2). Below, we address the mechanisms and functions of p38 MAPK signalling in IVDD.

### 5.1 Mediating inflammatory response

An inflammatory response is a crucial factor in the progression of IVDD. Degenerated IVD tissue contains inflammatory‐like cells that can spontaneously produce chemokines, including monocyte chemoattractant protein (MCP)‐1 and C‐C motif chemokine ligand 4 (CCL4). These chemokines can recruit macrophage infiltration that can release inflammatory cytokines to promote IVDD progression.^44^, ^45^ Moreover, several reports suggest that the activation of p38 MAPK signalling is associated with mediating the inflammatory response in IVDD. For example, Yang et al.^41^ indicated that the knockdown of p38α can completely suppress macrophage M1 polarization, and the downregulation of p38β or p38δ can partially inhibit macrophage M1 polarization. In addition, it is well known that macrophage M1 polarization has pro‐inflammatory functions.^46^ A recent study found that the expression of CCL4 was elevated in degenerated NP tissue and associated with macrophage infiltration. Mechanically, the expression level of CCL4 was regulated by p38 MAPK and NF‐κB, and using the inhibitors of p38 MAPK and NF‐κB can suppress the upregulation of CCL4 expression.^47^ Moreover, inhibiting the activation of p38 MAPK signalling suppresses the production of inflammatory cytokines. Park et al.^48^ found that the p38 MAPK signalling inhibitor, SB202190, can decrease the production of IL‐6, IL‐8 and TNF‐α and inhibit IVDD progression. Genistein, a type of protein tyrosine kinase inhibitor, has been proven to suppress the expression of IL‐1β and TNF‐α by inhibiting the p38 MAPK signalling.^49^ In addition, Studer et al.^16^ found that p38 MAPK signalling was involved in nitric oxide (NO), prostaglandin E2 (PGE2) and IL‐6 production. Inhibition of p38 MAPK was found to decrease PGE2 and IL‐6 accumulation. Overall, there is increasing evidence that activated p38 MAPK signalling can promote the inflammatory response. Therefore, inhibition of p38 MAPK may be a promising strategy for alleviating IVDD progression.

### 5.2 Increasing ECM degradation

In healthy IVD, the anabolism and catabolism of ECM are in balance. However, various adverse factors can result in the dysregulation of ECM homeostasis and induce IVDD. Accumulating evidence shows that activated p38 MAPK signalling can influence ECM degradation by regulating anabolic and catabolic enzymes. For example, Ge et al.^17^ showed that the use of IL‐10 could alleviate IVDD by inhibiting ECM degradation. Mechanistically, similar to the p38 MAPK inhibitor, SB202190, IL‐10 increased the expression of collagen II and aggrecan by inhibiting the activity of p38 MAPK. Moreover, resistin can increase the expression of disintegrin and metalloprotease with thrombospondin motif‐5 (ADAMTS‐5) to promote the progression of IVDD by activating p38 MAPK signalling. However, the presence of a p38 inhibitor (SB203580) decreased the level of ADAMTS‐5 and thus reduced ECM degradation.^50^ Moreover, activation of p38 MAPK signalling was also considered to mediate ECM degradation in the IVD of diabetic rats. Chen et al. showed that activation of p38 MAPK signalling upregulated the level of metalloproteinases (MMP3 and MMP13) and ADAMTSs (ADAMTS‐4 and ADAMTS‐5) but decreased the expression of tissue inhibitors of metalloproteinases (TIMPs) in the IVD tissue of diabetic rats.^43^ In addition, a recent study showed that the umbilical cord mesenchymal stem cell‐conditioned medium (MSC‐CM) can alleviate NP mesenchymal stem cells' (NPMSCs') degeneration via high glucose levels. Specifically, they found that the use of MSC‐CM inhibited the activation of p38 MAPK signalling to promote the levels of collagen II and aggrecan.^51^ Taken together, the above results suggest that inhibiting the activation of p38 MAPK signalling can promote the restoration of ECM homeostasis in IVDD.

### 5.3 Promoting apoptosis and senescence

IVD cell apoptosis is widely considered to play a crucial role in the process of IVDD. Excessive IVD cell apoptosis can accelerate the decrease in cell density and catabolism of the ECM. Therefore, a decreased rate of cell apoptosis could improve the progression of IVDD. In the past few years, growing evidence has demonstrated that the activation of p38 MAPK signalling is involved in IVD cell apoptosis. For example, IL‐1β and TNF‐α can induce NP cell apoptosis by promoting the expression of caveolin‐1 to activate Wnt/β‐catenin signalling. However, the p38 MAPK inhibitor can reverse IL‐1β or TNF‐α‐mediated apoptosis by inhibiting the activity of caveolin‐1/β‐catenin cox.^52^ Xu et al.^53^ found that the mechano growth factor inhibited the mechanical overload‐induced NP cell apoptosis in vitro and suppressed the activation of p38 MAPK signalling to decrease the level of apoptosis protein and increase the expression of anti‐apoptosis protein. Moreover, a recent study showed a new regulatory mechanism, in which the activation of p38 MAPK signalling promoted NP cell apoptosis. Jiang et al.^54^ reported that the overexpression of the ubiquitin protein ligase E3 component n‐recognin 3 (UBR3) promoted NP cell apoptosis by activating p38 MAPK signalling and stimulating dual‐specificity phosphatase 1 (DUSP1) ubiquitination. However, the overexpression of DUSP1 can reverse the effect of UBR3 overexpression by inhibiting p38 MAPK signalling. Certainly, some studies have shown that the activation of p38 MAPK signalling increased the rate of cell apoptosis in AF and CEP.^55^, ^56^

Similar to the effects of cell apoptosis, cell senescence has been regarded as an important mechanism in the process of IVDD. Pang et al.^57^ demonstrated that the activation of p38 MAPK signalling was involved in NP cell senescence. In senescent NP cells, the percentage of SA‐β‐galactosidase‐positive NP cells and the expression of senescence proteins (p53, p21 and p16) were positively correlated with the activity of p38 MAPK signalling. However, the p38 MAPK inhibitor, SB202190, inhibited the high‐magnitude compression‐induced cell senescence. A recent study by Zhao et al.^58^ reported that the use of lysyl oxidase (LOX) can reduce substrate stiffness‐induced NP cell senescence by inhibiting integrin β1‐p38 MAPK signalling. Furthermore, a cultivation environment of acidic pH (pH = 6.2) was proved to promote NP cell senescence by promoting the activation of p38 MAPK signalling.^59^ To summarize, the above results indicate that inhibiting the activation of p38 MAPK signalling can alleviate IVD cell apoptosis and senescence.

### 5.4 Suppressing proliferation

The dysregulation of IVD cell proliferation is also considered an indicator of IVDD. It has been reported that p38 MAPK signalling can regulate IVD cell proliferation. For example, Wu et al.^60^ revealed that IL‐1β could activate p38 MAPK signalling to decrease the expression of histone deacetylase 4 (HDAC4), which resulted in an aggravation of the inflammatory response and cell cycle arrest. However, the use of the p38 MAPK inhibitor, SD0006, can suppress inflammation and promote cell proliferation by restoring the expression of HDAC4 and inhibiting p38 MAPK signalling. In another report, IL‐2 was also found to inhibit cell proliferation by promoting the activation of p38 MAPK signalling.^61^ Moreover, the study showed that oxidative stress can lead to a G1 cell cycle delay and decrease cell proliferation by activating p38 MAPK signalling.^62^ Taken together, the activation of p38 MAPK signalling has a negative effect on IVD cell proliferation. However, the mechanism by which IVD cell proliferation regulates is still unclear, and further exploration is necessary.

### 5.5 Inhibiting autophagy

Autophagy is a conserved intracellular degradation process that maintains metabolism and homeostasis. In recent years, autophagy has been proven to play a crucial role in the progress and treatment of IVDD.^63^ As a major signal transduction pathway, p38 MAPK signalling is also considered to regulate the level of autophagy in IVDD. Zhang et al.^64^ demonstrated that quercetin can alleviate the progress of IVDD by preventing apoptosis and ECM degeneration. Mechanistically, the protective effect of quercetin was associated with the activation of autophagy. Further studies found that the inhibition of p38 MAPK activity can upregulate the level of autophagic flux by suppressing the mammalian target of rapamycin (mTOR) pathway. Hence, this evidence indicates that the activation of p38 MAPK can inhibit autophagy. Moreover, though p38 MAPK activity is confirmed to regulate autophagy, it is necessary to further explore the specific function of p38 MAPK in the process of autophagy.

## 6 TARGETING p38 MAPK SIGNALLING FOR THE TREATMENT OF IVDD

Over the past few years, developing p38 MAPK inhibitors has been a potential strategy for the treatment of multiple diseases, such as rheumatoid arthritis, chronic obstructive pulmonary disease and cancer. Certainly, the use of p38 MAPK inhibitors results in an obvious improvement in the prognosis and survival rates for some diseases. In recent years, targeting p38 MAPK by the application of p38 MAPK inhibitors has been shown to alleviate the progression of IVDD in vitro and in vivo **(**Table 1 **)**.

**TABLE 1:** Applications of p38 MAPK inhibitors in intervertebral disc degeneration (IVDD) treatment.

Compounds	Dosages	Mode of administration	Cell types	Animal model data	Effects	References
SD0006	70 nM	Incubation with SD0006	Rat NP cells	‐	Promoting NP cell proliferation and inhibiting ECM degeneration	60
SB202190	2 mM, 10 μM	Incubation with SB202190 and subcutaneous injection	Rat NP cells	Increasing T2 weighted signal	Suppressing NP apoptosis, ECM degeneration, senescence and inflammatory response	48, 65
SB203580	10 μM	Incubation with SB203580 and subcutaneous injection	Rat and rabbit NP cells	Increasing T2 weighted signal	Suppressing NP apoptosis, ECM degeneration, senescence and inflammatory response	57, 66, 67, 68, 69
Quercetin	25 μM, 100 mg/kg	Incubation with quercetin and administered intragastrically	Rat NP cells	Increasing T2 weighted signal and improving histopathological grading scores	Inhibiting NP apoptosis, ECM degeneration and restoring autophagy	64
Sodium tanshinone IIA	10, 20 mg/kg	Administered intragastrically	‐	Increasing T2 weighted signal and improving IVD volume	Inhibiting ECM degeneration and decreasing the IL‐6 and TNF‐α levels	42
IL‐10	10 ng/mL	Incubation with IL‐10 and subcutaneous injection	Rat NP cells	Increasing T2 weighted signal and decreasing Thompson scores	Inhibiting ECM degeneration	17
Tanshinone IIA	5 μM	Incubation with tanshinone IIA and subcutaneous injection	Rat NP cells	Inhibiting rat pain behaviour in vivo	Inhibiting inflammatory response and ECM degeneration	70
Genistein	30 ng/mL, 20 μg/mL	Incubation with genistein and subcutaneous injection	Rat NP cells	Increasing T2 weighted signal and decreasing Thompson scores	Inhibiting inflammatory response and ECM degeneration	49
Duhuo Jisheng decoction	200 μg/mL	Incubation with Duhuo Jisheng decoction and subcutaneous injection	Rat NP cells	Increasing T2 weighted signal and decreasing Pfirrmann scores	Inhibiting NP cell apoptosis and ECM degeneration	71
Mechano growth factor	40 ng/mL	Incubation with mechano growth factor	Rat NP cells	‐	Alleviating NP cell apoptosis	53

Abbreviations: ECM, extracellular matrix; IL, interleukin; IVD, intervertebral disc; NP, nucleus pulposus; TNF‐α, tumour necrosis factor‐alpha.

### 6.1 p38 MAPK inhibitors

At present, p38 MAPK inhibitors are only used in disease models for the IVDD treatment because of their limitations in safety and method of use. For example, SB202190, as a p38 MAPK inhibitor, has been found to inhibit the inflammatory response and ECM degradation to degenerate NP cells.^16^, ^48^ SB203580 is another p38 MAPK inhibitor that was found to decrease the rate of cell apoptosis and inhibit the production of inflammatory cytokines, which, in turn, alleviated the progression of IVDD in a rat IVDD model.^47^, ^66^, ^72^, ^73^ A recent study showed that another p38 MAPK inhibitor, SD0006, could also inhibit the inflammatory response and promote NP cell proliferation by regulating the p38 MAPK/HDAC4 pathway.^60^ Moreover, genistein, as a protein tyrosine kinase inhibitor, has been identified to inhibit the inflammatory response and delay the process of IVDD by inhibiting p38 MAPK.^49^ In addition to specific p38 MAPK inhibitors, there are some traditional Chinese medicine (TCM) and natural compounds that have been found to alleviate IVDD by suppressing p38 MAPK. Duhuo Jisheng decoction is a typical TCM prescription and has been used in the treatment of multiple diseases. A recent study showed that Duhuo Jisheng decoction could reduce NP cell apoptosis and ECM degradation by inhibiting p38 MAPK signalling in a rat IVDD model.^71^ As a natural compound extracted from garlic bulbs, allicin was demonstrated to decrease oxidative stress and mitochondrial apoptosis by inhibiting the activation of p38 MAPK signalling.^74^ Moreover, a recent report found that sodium tanshinone IIA sulfonate extracted from Danshen could reduce oxidative stress and alleviate the progression of IVDD by inhibiting the activity of p38 MAPK signalling.^42^ Overall, compared with specific p38 MAPK inhibitors, the protective roles of other inhibitors, such as natural compounds, are also effective in the IVDD treatment.

### 6.2 Targeting upstream regulators or downstream targets of p38 MAPK

In addition to p38 MAPK inhibitors, targeting upstream regulators or downstream targets of p38 MAPK is a promising strategy for the IVDD treatment. For example, Cui and Zhang^15^ showed that the 3′‐untranslated region (3′‐UTR) of leucine‐rich α2‐glycoprotein 1 (LRG1) was involved in the activation of p38 MAPK signalling. They found that the overexpression of LRG1 could increase the p‐p38/p38 rate, and the effect of downregulation of LRG1 was similar to that of the p38 MAPK inhibitor SB202190 on the activity of p38 MAPK. Thereby, this indicates that LRG1 may be an upstream regulator of p38 MAPK in IVDD. Moreover, dual‐specificity (Thr/Tyr) phosphatase 1 (DUSP1) was identified to promote the dephosphorylation of p38 MAPK. In a study by Jiang et al.,^54^ overexpression of DUSP1 not only considerably promoted the dephosphorylation of p38 MAPK in the vector group but also reversed the phosphorylation of p38 MAPK in the UBR3 overexpression group. Certainly, the above results only provide indirect evidence regarding the upstream regulators of p38 MAPK. Meanwhile, there are still few studies directly associated with the upstream regulators or downstream targets of p38 MAPK. Therefore, further exploration of the upstream regulators and downstream targets of p38 MAPK, including MKK3, MKK4 and MKK6 and the downstream targets MAPKAPK2 (MK2), is required.

## 7 CONCLUSION

IVDD is a complex pathological process mediated by multiple factors, including ageing, mechanical overloading, genetics, environment and injury. Therefore, it is necessary to explore new mechanisms and develop promising targets for the IVDD treatment. As mentioned above, the activation of p38 MAPK signalling can contribute to the progression of IVDD, including mediating inflammatory response, increasing ECM degradation, promoting apoptosis and senescence and suppressing proliferation and autophagy. Meanwhile, increasing reports show that targeting p38 MAPK signalling can alleviate IVDD progression. However, applications targeting p38 MAPK signalling have only been tested in various animal or cell models, and there is no evidence to show their safety and effect in clinical trials. Therefore, future studies should focus on solving the following issues: first, exploring the specific functions of upstream regulators or downstream targets of p38 MAPK in IVDD is a pressing matter. Moreover, the triggers for the dysregulation of p38 MAPK signalling and mechanisms to restore the dysregulation of p38 MAPK signalling remain unknown in the process of IVDD.

## AUTHOR CONTRIBUTIONS

Ding‐jun Hao was responsible for the conception, design and final approval. Zheng‐wei Shi and Lei Zhu performed searches and analyses and drafted the manuscript. Zong‐rang Song and Tuan‐jiang Liu took part in revising the grammar. All the authors read and approved the final manuscript.

## CONFLICT OF INTEREST STATEMENT

The authors declare no conflicts of interest.
